# Variability of Nutritional Status During Hematopoietic Stem Cell Transplantation and Its Association With Clinical Outcomes: An Ambispective Observational Study From India

**DOI:** 10.7759/cureus.116091

**Published:** 2026-09-11

**Authors:** Manasi Gupta, Himanshu Aggarwal, Ruma Singh, Vijay Chaudhary, Pavitra D S, Chirag Trivedi, Shruti Kakkar, Ashwin Philips, M Joseph John

**Affiliations:** 1 Department of Clinical Haematology, Haemato-Oncology, and Bone Marrow (Stem Cell) Transplantation, Christian Medical College Ludhiana, Ludhiana, IND; 2 Department of Dietetics, Christian Medical College & Hospital, Ludhiana, Ludhiana, IND; 3 Department of Haematology and Medical Oncology, Kasturba Medical College, Manipal, Manipal, IND; 4 Department of Medical Oncology, Christian Medical College Ludhiana, Ludhiana, IND; 5 Department of Clinical Haematology, Haemato-Oncology, and Bone Marrow (Stem Cell) Transplantation, Christian Medical College & Hospital, Ludhiana, Ludhiana, IND

**Keywords:** calorie intake, hematopoietic stem cell transplantation, india, infection, malnutrition, nutrition, quality of life, transplant outcomes, weight loss

## Abstract

Background

Nutritional deterioration is frequent during hematopoietic stem cell transplant (HSCT), but Indian data detailing serial weight and calorie intake changes and their outcome associations remain limited. This study evaluated peri-transplant nutritional variability among HSCT recipients at an Indian tertiary care center, exploring relationships with infectious morbidity, hospitalization, quality of life, nonrelapse mortality, and survival.

Methods

We conducted an ambispective observational cohort study of 50 HSCT recipients (26 prospective and 24 retrospective) treated between December 2021 and October 2023. Serial nutritional assessments included weight, body mass index or pediatric anthropometry, calorie intake, and biochemical parameters at baseline and post-transplant time points. Outcomes evaluated were mucositis, acute graft-versus-host disease, infections (bacterial, viral, and fungal), quality of life, length of hospital stay, non-relapse mortality, and overall survival. Statistical evaluations utilized repeated-measures and univariable analyses, Kaplan-Meier survival estimations, and a linear mixed-effects model for post-transplant weight trajectories.

Results

The median age was 19.5 years (range: 2-64), with 29 males. Transplants were allogeneic (n = 36) or autologous (n = 14), and baseline nutritional abnormalities (under/overnutrition) were present in 19 patients. Mean weight change from admission progressed from -1.80% at day 0 to -7.65% at day +30 and -7.60% at day +60. Mean calorie intake declined most sharply by day +7 (-29.0%), improving gradually thereafter. At day +60, mean weight loss was greater in patients with viral infections (-14.7% vs. -5.4%; p = 0.03) and hospital stays > 36 days (-14.6% vs. -5.3%; p = 0.025). Reduced calorie intake was associated with bacterial infections at days 0, +21, and +60. Quality-of-life scores declined post-transplant, partially recovering by day +60, and day +100 non-relapse mortality was 16%. At a median 340-day follow-up, overall survival was 68%, and the estimated one-year overall survival probability was 72% (95% confidence interval, 60%-82%). In the linear mixed-effects model, age, sex, and diagnosis did not significantly predict weight change. Marginal (0.066) and conditional (0.680) R-squared values showed repeated-measures structures captured greater observed variability than fixed demographic/diagnostic covariates.

Conclusion

Peri-transplant nutritional decline was common in this Indian HSCT cohort. Weight loss continued beyond maximal calorie reduction, associating with adverse outcomes like viral infections and prolonged hospitalization, whereas reduced calorie intake correlated with bacterial infections. These findings support structured nutritional assessments, early dietetic involvement, and proactive nutritional support in routine transplant care. Larger prospective multicenter studies are needed to confirm associations and define standardized nutrition-focused HSCT interventions.

## Introduction

Hematopoietic stem cell transplant (HSCT) is an established treatment for a wide range of malignant and non-malignant hematologic disorders. The transplant process, however, exposes patients to substantial metabolic and inflammatory stress. Conditioning chemotherapy, radiotherapy in selected settings, mucositis, infections, nausea, vomiting, diarrhea, dysgeusia, graft-versus-host disease (GVHD), corticosteroid exposure, and prolonged hospitalization can reduce oral intake while increasing catabolic demand. These factors make nutritional decline a common and clinically important problem in HSCT recipients [1-3].

Pre-transplant nutritional vulnerability may be particularly relevant in India. Indian transplant cohorts often include children, adolescents, and young adults with transfusion-dependent thalassemia, aplastic anemia, and other chronic hematologic conditions. Nutritional impairment among children with beta-thalassemia major has been described in Indian settings and may reflect chronic disease burden, iron overload, socioeconomic constraints, endocrine complications, and reduced growth reserve [4]. Patients with hematologic malignancies may also enter transplant after multiple cycles of intensive therapy, inflammatory stress, poor appetite, and reduced functional reserve [2].

Nutritional assessment in HSCT is clinically necessary but difficult to interpret. Weight and body mass index may be influenced by hydration, edema, fluid shifts, inflammation, gastrointestinal toxicity, corticosteroid therapy, and changes in muscle mass. Serum albumin is affected by inflammation and capillary leak and therefore cannot be considered a nutritional marker in isolation. Serial assessment is therefore more informative than a single baseline measurement. International transplant nutrition literature supports systematic nutritional support, early dietetic involvement, and timely escalation to enteral or parenteral nutrition when oral intake remains inadequate [5].

Despite the clinical importance of nutrition in HSCT, Indian data on serial peri-transplant weight and calorie-intake trajectories remain limited. Therefore, the primary objective of this study was to characterize serial changes in body weight and calorie intake from admission through day +60 among patients undergoing hematopoietic stem cell transplantation at an Indian tertiary-care center. The secondary exploratory objective was to examine whether these nutritional changes are associated with mucositis, acute graft-versus-host disease, bacterial, viral, and fungal infections, quality of life, duration of hospitalization, day +100 non-relapse mortality, and overall survival.

## Materials and methods

Study design and setting

This was an ambispective observational cohort study conducted in the Department of Clinical Haematology, Haemato-Oncology, and Bone Marrow Transplantation and the Department of Dietetics at Christian Medical College & Hospital, Ludhiana, India. The study period extended from December 2021 to October 2023. The retrospective cohort included patients transplanted from December 2021 to November 2022, and the prospective cohort included patients enrolled from November 2022 to October 2023.

Ethics and registration

The study was reviewed and approved by the Institutional Ethics Committee of Christian Medical College, Ludhiana, India. The IEC approval reference number was BMHR-IECCMCL/1122-416/ApprvI-DM-Thesis/Clnl-Haemat. The IEC registration number was ECR/120/Inst/PB/2013 (issued under Rule 122DD). The study was registered with the Clinical Trials Registry-India as CTRI/2022/12/048066. Written informed consent was obtained from prospectively enrolled participants or their legally acceptable representatives. Retrospective data were abstracted from hospital records according to institutional approval and confidentiality requirements. No patient-identifying information was used in the analysis or manuscript.

Participants

All patients undergoing HSCT during the study period were eligible, irrespective of age, sex, underlying diagnosis, transplant type, and comorbidity profile. Refusal to consent was the only exclusion criterion for the prospective component. The final cohort included 50 patients, comprising 26 prospectively enrolled patients and 24 retrospectively reviewed patients. Participant recruitment, cohort allocation, and serial data availability are summarized in Figure 1.

**Figure 1:**
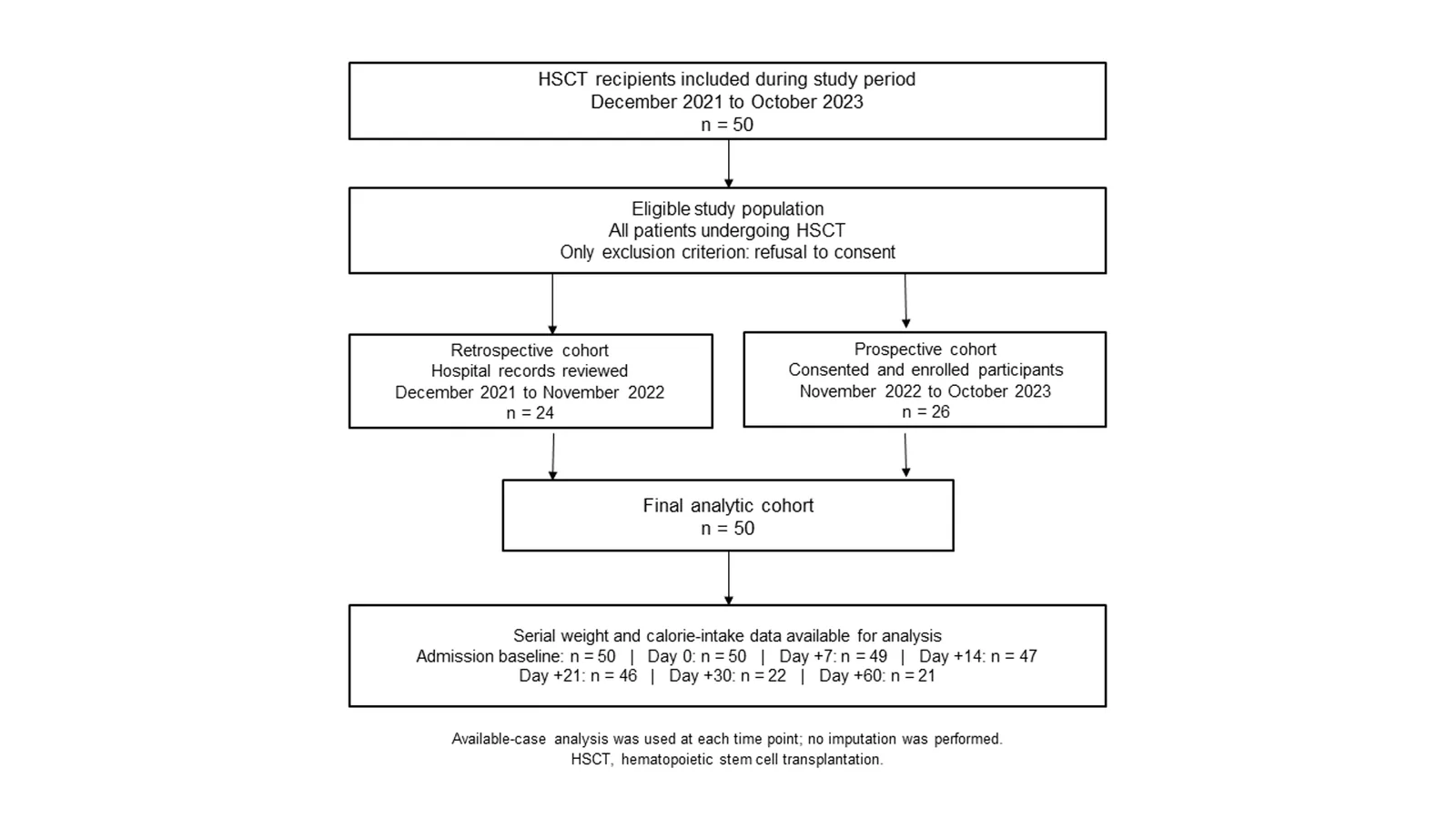
Participant recruitment and data availability flow The figure summarizes the ambispective study cohort of 50 HSCT recipients included between December 2021 and October 2023. The retrospective cohort included 24 patients, and the prospective cohort included 26 patients. The final analytic cohort included 50 patients. Serial weight and calorie-intake data were available for 50 patients at admission baseline, 50 at day 0, 49 at day +7, 47 at day +14, 46 at day +21, 22 at day +30, and 21 at day +60. Available-case analysis was used at each time point, and no imputation was performed. HSCT: Hematopoietic stem cell transplantation.

Study size

The planned sample size was 50 patients. In the thesis protocol, this was based on an expected pre- and post-admission calorie-intake difference of 628 kcal, with a type I error of 0.05 and type II error of 0.20. For the final manuscript analyses, the study was treated as an exploratory ambispective cohort because of its single-center design, heterogeneous transplant population, and limited number of later post-transplant observations.

Clinical and nutritional assessment

Baseline data included age, sex, primary diagnosis, transplant type, donor category when applicable, conditioning regimen, performance status, hematopoietic cell transplantation-specific comorbidity index (HCT-CI), and relevant laboratory parameters [6]. Nutritional assessment included weight, adult body mass index, available pediatric anthropometry, calorie intake, serum albumin, hemoglobin, and creatinine. For adults, body mass index (BMI) was categorized as underweight (<18.5 kg/m²), normal (18.5-24.9 kg/m²), overweight (25.0-29.9 kg/m²), and obesity (≥30 kg/m²). For pediatric and adolescent patients, nutritional classification was based on age- and sex-appropriate anthropometric assessments using standard World Health Organization (WHO) growth reference charts documented in clinical and dietetic records, including BMI-for-age and/or mid-upper arm circumference when available. When the medical record contained a dietitian-assigned nutritional category rather than raw z-scores, that category was retained. For analysis, nutritional categories were harmonized as underweight, normal, overweight, and obese. In descriptive analyses, nutritional abnormality referred to patients outside the normal category, including both undernutrition and overnutrition.

Calorie intake was estimated using 24-hour dietary recall at admission and available dietetic records or recall-based assessment for subsequent time points. The Indian Council of Medical Research-National Institute of Nutrition dietary tables were used to support calorie calculations [7]. Baseline assessment was performed after admission to the bone marrow transplant unit before conditioning. Subsequent planned time points were day 0, day +7, day +14, day +21, day +30, and day +60. Day 0 was defined as the day of stem cell infusion. For post-admission time points, calorie intake reflected the average intake during the preceding week when available. For the prospective arm, data collected at day +28 plus or minus three days were represented as day +30. For retrospective patients, data were abstracted from Medical Records Department files and available dietetic or clinical documentation. The clinical and nutritional assessment schedule is summarized in Figure 2.

**Figure 2:**
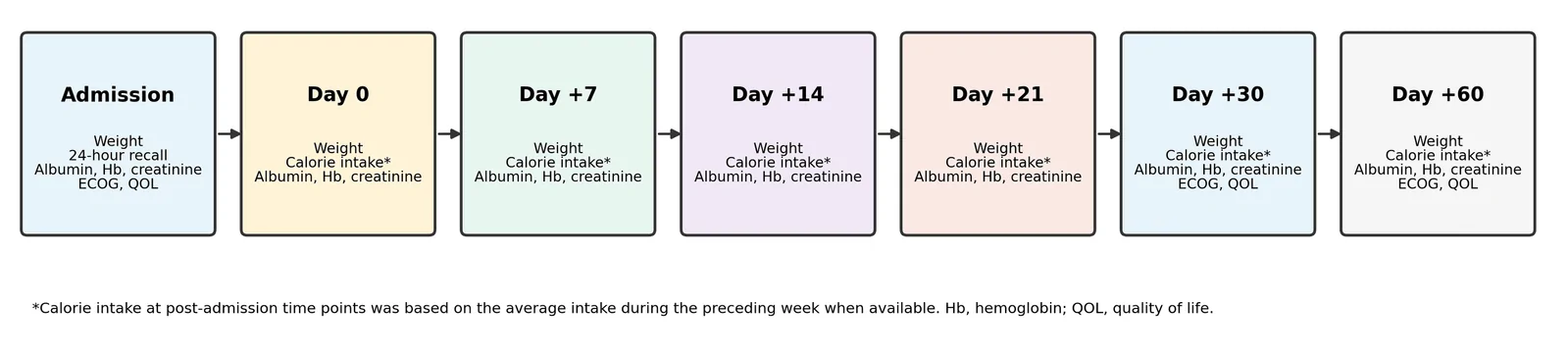
Schedule of clinical and nutritional assessments The figure shows the planned clinical and nutritional assessment schedule from admission baseline through day 0, day +7, day +14, day +21, day +30, and day +60. Admission baseline included weight, 24-hour dietary recall, albumin, hemoglobin, creatinine, ECOG performance status, and quality-of-life assessment when available. Post-admission calorie intake reflected the average intake over the preceding week when available. Hb: Hemoglobin; QOL: Quality of life; ECOG: Eastern Cooperative Oncology Group.

Outcomes and definitions

The outcomes of interest were mucositis, acute GVHD, bacterial infection, viral infection, fungal infection, quality of life, length of hospital stay, non-relapse mortality, and overall survival. Mucositis was graded using the National Cancer Institute Common Terminology Criteria for Adverse Events, version 5.0 [8]. Acute GVHD was assessed using Mount Sinai Acute GVHD International Consortium criteria [9]. Quality of life was assessed using the European Organization for Research and Treatment of Cancer QLQ-C30 instrument [10]. Infections were classified from clinical documentation supported by microbiological, virological, fungal biomarker, culture, or radiological evidence when available. Overall survival was calculated from the date of transplant to death from any cause or last follow-up. Non-relapse mortality was assessed at day +100.

Statistical analysis

Continuous variables were summarized as mean with standard deviation or median with range, depending on distribution and reporting context. Categorical variables were summarized as frequencies and percentages. Serial changes in weight and calorie intake were expressed as mean percentage change from admission baseline. The mean change line figure begins at admission baseline, where both weight and calorie-intake percentage change are set at 0.0%, and then displays subsequent mean percentage changes at day 0, day +7, day +14, day +21, day +30, and day +60.

Repeated-measures analyses were used to evaluate within-patient trends across predefined transplant time points. Sphericity was assessed using Mauchly's test. When sphericity was violated, Greenhouse-Geisser or Huynh-Feldt corrections were applied, and Bonferroni correction was used for post hoc comparisons. Univariable associations between nutritional variables and clinical outcomes were examined using appropriate group comparison tests for continuous variables and chi-square or Fisher's exact tests for categorical variables. Exploratory subgroup comparisons were performed by age group, diagnosis category, transplant type, and baseline nutritional category.

Kaplan-Meier analysis was used to estimate overall survival, and survival curves were compared using the log-rank test. A linear mixed-effects model was used to evaluate percentage change in post-transplant weight while accounting for repeated observations within patients and variation across time points. Fixed effects included age, sex, and primary diagnosis, with thalassemia used as the reference diagnosis. Random effects accounted for patient-level and day-level clustering. Model estimates were reported with standard errors, 95% confidence intervals, p-values, variance components, intraclass correlation coefficient, marginal R-squared, and conditional R-squared. Missing values were not imputed; analyses used available observations at each time point. No formal sensitivity analyses were performed. Because of the sample size, clinical heterogeneity, and multiple exploratory comparisons, outcome association analyses were interpreted as hypothesis-generating rather than confirmatory. A two-sided p-value less than 0.05 was considered statistically significant. Analyses were performed using IBM SPSS Statistics version 20 (IBM Corp., Armonk, NY) and R version 4.5.0 (R Foundation for Statistical Computing, Vienna, Austria).

Reporting of this study followed the STROBE (Strengthening the Reporting of Observational Studies in Epidemiology) guidelines for cohort studies [11].

## Results

Patient characteristics

Fifty HSCT recipients were included. The median age was 19.5 years, with a range of 2-64 years. Twenty-nine patients were male, and 21 were female. Thirty-six patients underwent allogeneic transplant, and 14 underwent autologous transplant. The most common primary diagnoses were transfusion-dependent thalassemia major in 20 patients, multiple myeloma in 10 patients, and aplastic anemia in eight patients. Baseline characteristics are shown in Table 1.

**Table 1:** Baseline characteristics of the study cohort Categorical variables are presented as n (%). Continuous variables are presented as median (lower quartile-upper quartile). AML: Acute myeloid leukemia; MPN: Myeloproliferative neoplasm; NHL: Non-Hodgkin lymphoma.

Characteristic	Value
Age, years	19.5 (9.0-40.5)
Sex	
Male	29 (58%)
Female	21 (42%)
Transplant type	
Allogeneic transplant	36 (72%)
Autologous transplant	14 (28%)
Primary diagnosis	
Thalassemia major	20 (40%)
Aplastic anemia	8 (16%)
Acute myeloid leukemia	5 (10%)
Myeloproliferative neoplasm	3 (6%)
Multiple myeloma	10 (20%)
Hodgkin lymphoma	3 (6%)
Non-Hodgkin lymphoma	1 (2%)
Baseline nutritional category	
Normal	31 (62%)
Underweight	4 (8%)
Overweight	7 (14%)
Obese	8 (16%)

Baseline nutritional status

At baseline, 31 patients were classified as nutritionally normal, four were underweight, seven were overweight, and eight were obese. Thus, 19 patients had nutritional abnormality when undernutrition and overnutrition were considered together. Overweight and obesity were more frequent among adults and patients with multiple myeloma, whereas undernutrition was more common among patients with thalassemia. These patterns reflected the clinical heterogeneity of the transplant population, which included pediatric and adult patients, benign and malignant disorders, and both allogeneic and autologous transplants.

Serial changes in weight and calorie intake

Mean weight change showed progressive decline after admission baseline. Mean percentage weight change was -1.80% at day 0, -3.44% at day +7, -5.11% at day +14, -6.87% at day +21, -7.65% at day +30, and -7.60% at day +60. Mean calorie intake showed its greatest early decline at day +7, with a mean absolute change of -441 kcal and a mean percentage change of -29.0%, followed by gradual improvement by day +60. Repeated-measures ANOVA showed a significant change in percentage weight change across serial time points (F(6,120) = 13.68, p < 0.001) and a significant early change in percentage calorie-intake change from admission baseline through day +14 (F(3,141) = 18.09, p < 0.001). The serial trends are summarized in Table 2 and shown in Figure 3.

**Table 2:** Serial trends in weight and calorie intake during and after transplantation Values are presented in mean ± SD format unless otherwise indicated. Negative values indicate a decline from the admission baseline. Admission baseline was set at 0.0% for the mean change trajectory. Day 0 refers to the day of stem cell infusion.

Time point	Admission baseline	Day 0	Day +7	Day +14	Day +21	Day +30	Day +60
n	50	50	49	47	46	22	21
Weight change from admission baseline, %, mean ± SD	0.00 ± 0.00	-1.80 ± 4.34	-3.44 ± 5.05	-5.11 ± 5.46	-6.87 ± 5.11	-7.65 ± 6.48	-7.60 ± 8.32
Calorie change from admission baseline, kcal, mean ± SD	0.00 ± 0.00	-335 ± 302	-441 ± 418	-389 ± 455	-229 ± 535	-209 ± 656	-78.9 ± 512
Calorie change from admission baseline, %, mean ± SD	0.00 ± 0.00	-22.6 ± 21.4	-29.0 ± 28.2	-21.4 ± 38.0	-7.54 ± 47.4	-5.15 ± 47.1	2.59 ± 37.9

**Figure 3:**
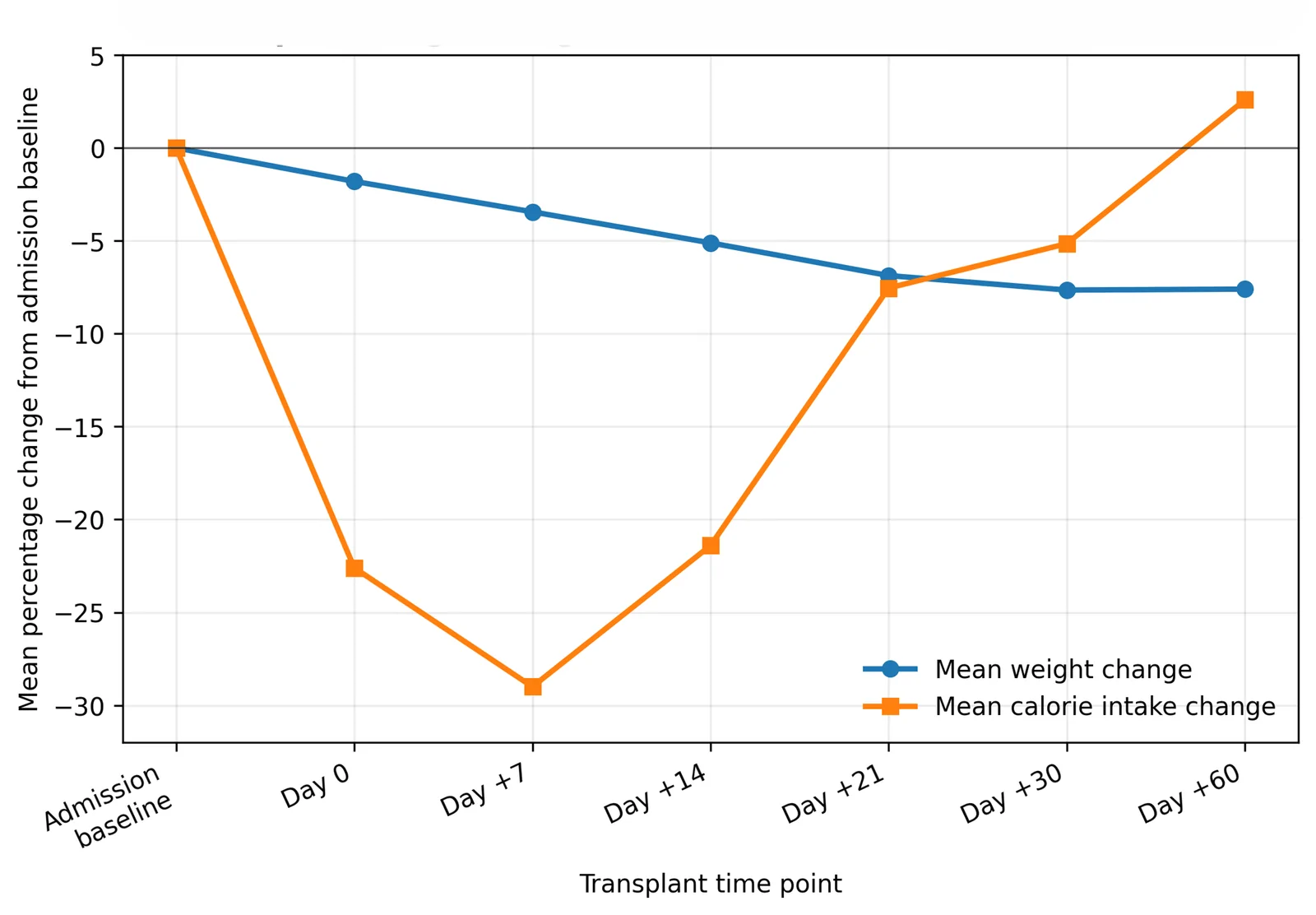
Mean percentage change in weight and calorie intake across transplant time points The line figure displays group-level mean percentage change in weight and calorie intake across serial transplant time points. The number of patients at each time point was as follows: admission baseline, n = 50; day 0, n = 50; day +7, n = 49; day +14, n = 47; day +21, n = 46; day +30, n = 22; and day +60, n = 21. The figure begins at admission baseline with both weight and calorie-intake lines set at 0.0% because all subsequent points represent mean percentage change from admission baseline. Day 0 refers to the day of stem cell infusion and is plotted after admission baseline. The weight trajectory shows progressive decline through day +30 and persistence to day +60, while calorie intake shows the largest early decline at day +7 followed by gradual recovery. This figure shows group-level descriptive trajectories and should not be interpreted as a patient-level correlation between calorie intake and weight change.

The number of available observations decreased at day +30 and day +60 because many patients had been discharged from the transplant unit by these time points, and several retrospective records had incomplete later dietetic or anthropometric documentation. Early mortality and clinical instability also contributed to the non-availability of measurements in a small number of patients. Consequently, day +30 and day +60 estimates represent available-case observations and should be interpreted cautiously.

Transplant-related complications and clinical outcomes

Mucositis occurred in 18 patients, mostly as grade 1 disease. Acute GVHD occurred in 19 of the 36 patients who underwent allogeneic transplant, with gastrointestinal involvement being the predominant pattern. Bacterial infections occurred in 31 patients, viral infections in 16 patients, and fungal infections in 15 patients. *Klebsiella *species were the most commonly documented bacterial pathogens, and cytomegalovirus was the most common viral infection. Quality-of-life scores declined after transplant, reached the lowest observed values around day +30, and partially improved by day +60 without returning fully to baseline. Day +100 non-relapse mortality was 16%. At a median follow-up of 340 days, overall survival was 68%, and the estimated one-year overall survival probability was 72% (95% confidence interval, 60%-82%). Median survival was not reached. Overall survival is shown in Figure 4.

**Figure 4:**
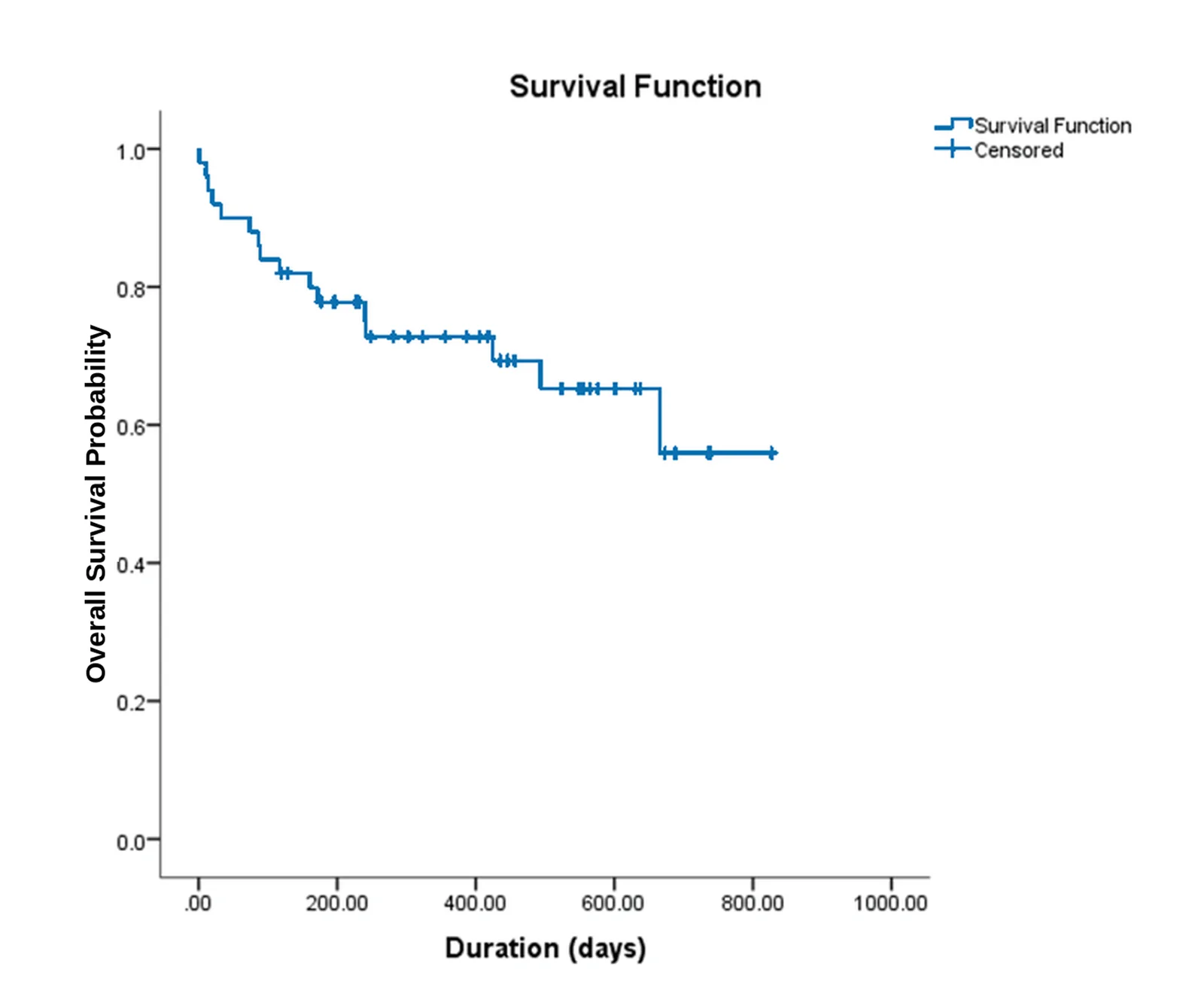
Kaplan-Meier curve for overall survival Overall survival was calculated from the date of transplant to death from any cause or last follow-up. At a median follow-up of 340 days, overall survival was 68%, and the estimated one-year overall survival probability was 72%, with a 95% confidence interval of 60%-82%. Median survival was not reached.

Survival did not differ significantly across baseline nutritional categories, as shown in Figure 5, but this analysis was limited by small subgroup sizes, particularly among obese and underweight patients, and a type II error cannot be excluded. Main clinical outcomes are summarized in Table 3.

**Figure 5:**
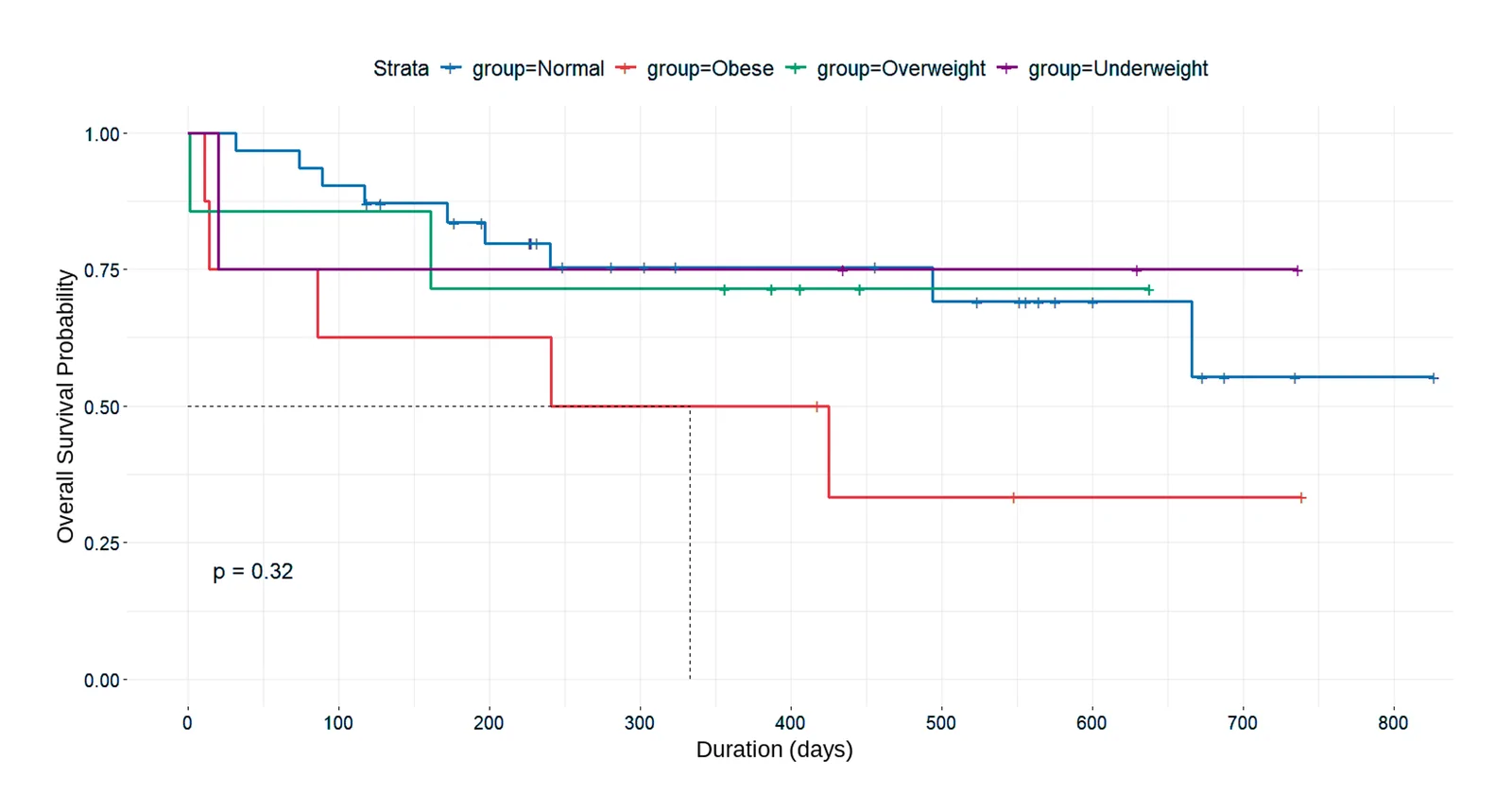
Kaplan-Meier curves for overall survival stratified by baseline nutritional category Kaplan-Meier curves show overall survival stratified by baseline nutritional category. Survival did not differ significantly across baseline nutritional categories in this cohort. The p-value was calculated using the log-rank test. The obese and underweight groups are displayed with distinctly different colors to improve visual discrimination. Interpretation should be cautious because subgroup sizes were small, particularly among obese and underweight patients, and a type II error cannot be excluded.

**Table 3:** Main transplant-related complications and clinical outcomes Percentages are based on the full cohort unless otherwise indicated. Acute GVHD percentage is based on allogeneic transplant recipients only. GVHD: Graft-versus-host disease; CI: Confidence interval.

Outcome	n/N	% or estimate
Mucositis	18/50	36%
Acute GVHD among allogeneic recipients	19/36	52.80%
Bacterial infection	31/50	62%
Viral infection	16/50	32%
Fungal infection	15/50	30%
Day +100 non-relapse mortality	8/50	16%
Overall survival at median follow-up	34/50	68%
Estimated one-year overall survival	-	72% (95% CI, 60%-82%)

Associations between nutritional change and clinical outcomes

In the available-case analysis at day +60, patients with viral infections had greater mean weight loss than those without viral infections (-14.7% versus -5.4%; independent-samples t-test, t(19) = 2.43, p = 0.025). Patients with hospital stay longer than 36 days also had greater mean weight loss than those with shorter hospital stay (-14.6% versus -5.3%; independent-samples t-test, t(19) = 2.44, p = 0.025). These day +60 comparisons were based on small exposed subgroups, with five patients in the viral infection group and five patients in the prolonged-stay group, and should therefore be considered exploratory signals rather than definitive evidence. Reduced calorie intake was associated with bacterial infections at day 0 (t(48) = 3.69, p < 0.001), day +21 (t(44) = 3.17, p = 0.003), and day +60 (t(19) = 2.53, p = 0.020). The selected exploratory statistical results are presented in Table 4. These findings should be interpreted as associations rather than causal effects because infection, mucositis, GVHD, systemic inflammation, gastrointestinal symptoms, and reduced oral intake may influence each other bidirectionally.

**Table 4:** Exploratory associations between nutritional change and selected clinical outcomes Values are exploratory univariable comparisons and should not be interpreted as causal effects. Mean changes are presented as mean ± SD and represent percentage changes from admission baseline. Group-specific sample sizes are shown within the comparison-group columns. Statistical comparisons were performed using independent-samples Student’s t-tests; test statistics are reported as t(df). p-values are two-sided.

Nutritional variable/time point	Outcome comparison	First comparison group, n; mean change (%), mean ± SD	Second comparison group, n; mean change (%), mean ± SD	Statistical test and test statistic	p-value
Weight change, day +60	Viral infection	No viral infection, n = 16; -5.4 ± 6.7	Viral infection, n = 5; -14.7 ± 9.8	Independent-samples t-test; t(19) = 2.43	0.025
Weight change, day +60	Hospital stay	≤36 days, n = 16; -5.3 ± 6.7	>36 days, n = 5; -14.6 ± 9.7	Independent-samples t-test; t(19) = 2.44	0.025
Calorie-intake change, day 0	Bacterial infection	No bacterial infection, n = 19; -9.9 ± 23.3	Bacterial infection, n = 31; -30.4 ± 16.0	Independent-samples t-test; t(48) = 3.69	<0.001
Calorie-intake change, day +21	Bacterial infection	No bacterial infection, n = 19; 16.5 ± 56.2	Bacterial infection, n = 27; -24.5 ± 31.3	Independent-samples t-test; t(44) = 3.17	0.003
Calorie-intake change, day +60	Bacterial infection	No bacterial infection, n = 8; 26.2 ± 40.5	Bacterial infection, n = 13; -12.0 ± 28.8	Independent-samples t-test; t(19) = 2.53	0.02

Linear mixed-effects model for post-transplant weight

In the linear mixed-effects model for percentage change in post-transplant weight, age, sex, and primary diagnosis were not statistically significant fixed-effect predictors of weight trajectory. Compared with thalassemia as the reference diagnosis, the diagnostic categories had confidence intervals that crossed zero. The model therefore did not identify a specific demographic or diagnostic group as the dominant driver of post-transplant weight decline. The marginal R-squared value was 0.066, indicating that the measured fixed effects explained only a small proportion of the variability in percentage weight change. The conditional R-squared value was 0.680, indicating that the full model, including patient-level and day-level random intercepts, accounted for a substantially larger proportion of variation. The intraclass correlation coefficient was 0.66, consistent with substantial clustering of repeated weight-change observations within the patient-time structure. Residual and quantile-quantile plot review did not suggest major departure from model assumptions. These findings support the interpretation that post-transplant weight loss was a common repeated-measures phenomenon in this cohort and was not explained solely by age, sex, or diagnosis. Fixed-effect model results are shown in Table 5, and the random-effects and model-fit summary is shown in Table 6.

**Table 5:** Linear mixed-effects model fixed-effect coefficients for percentage change in post-transplant weight Thalassemia was used as the reference diagnosis. Female sex was the reference category for the sex variable; therefore, the male coefficient is interpreted relative to females. t-statistics were calculated as the estimate divided by the standard error. Empty cells indicate the reference category.

Predictor	Estimate	Standard error	t-statistic	95% confidence interval	p-value	df
Intercept	-4.17	1.65	-2.53	-7.57 to -0.77	0.012	272
Age	0.09	0.07	1.29	-0.05 to 0.23	0.194	272
Male	-1.03	1.2	-0.86	-3.45 to 1.40	0.394	272
Primary diagnosis: thalassemia	Reference					
Acute myeloid leukemia	-3.5	2.3	-1.52	-8.16 to 1.15	0.129	272
Aplastic anemia	-1.1	2.42	-0.45	-5.99 to 3.79	0.650	272
Chronic myeloid leukemia	-4.71	3.34	-1.41	-11.46 to 2.03	0.159	272
Hodgkin lymphoma	-1.75	2.65	-0.66	-7.09 to 3.60	0.509	272
Myeloproliferative neoplasm	0.7	4.61	0.15	-8.63 to 10.03	0.879	272
Multiple myeloma	-5.37	3.47	-1.55	-12.38 to 1.64	0.123	272
Non-Hodgkin lymphoma	-4.46	4.94	-0.9	-14.39 to 5.47	0.367	272

**Table 6:** Random-effects structure and model-fit statistics for the linear mixed-effects model The model included fixed effects for age, sex, and primary diagnosis, with random intercepts for patient and day/time point to account for repeated measurements and time-point clustering.

Model component/fit statistic	Value
Random-effects structure	Random intercepts for patient and day/time point
Residual variance	11.24
Patient-level variance	13.87
Day-level variance	7.75
Intraclass correlation coefficient	0.66
Number of day levels	7
Number of patients	50
Observations	285
Marginal R-squared	0.066
Conditional R-squared	0.68

## Discussion

This ambispective Indian cohort study describes serial nutritional change during HSCT and its relationship with clinically relevant transplant outcomes. The central finding was that weight loss progressed after transplant and persisted to day +60, whereas calorie intake declined most sharply early after transplant and then improved gradually. This divergence between weight and calorie trajectories is clinically important because ongoing post-transplant weight loss may reflect more than simple calorie deficit. Inflammation, infection, GVHD, gastrointestinal toxicity, catabolism, fluid shifts, treatment-related metabolic stress, and reduced functional reserve are all plausible contributors.

The findings are consistent with international literature showing that weight loss after HSCT is common and may exceed five percent in many recipients [12,13]. They also align with reports emphasizing the importance of structured nutritional rehabilitation before and during transplant. A multicenter survey from the United Kingdom found that transplant professionals recognized the value of nutritional prehabilitation, but formal pre-transplant rehabilitation pathways were available in only a minority of centers [14]. Recent work also suggests that energy expenditure may increase after HSCT, further supporting the need for careful nutritional surveillance during the peri-transplant period [15].

The Indian context gives this study additional relevance. The cohort included a substantial proportion of children and young adults with thalassemia, as well as adults with multiple myeloma and other hematologic malignancies. This combination of benign and malignant indications reflects the real-world case mix of many Indian transplant units. Nutritional vulnerability may differ from Western cohorts because of younger transplant age, chronic transfusion-dependent disorders, pre-existing growth or nutritional deficits, socioeconomic barriers, infection burden, and variability in access to structured dietetic follow-up. The observation that undernutrition was more frequent in thalassemia while overweight and obesity were more common in older adults and patients with myeloma underscores the need to avoid treating malnutrition as a single biological state. Both undernutrition and overnutrition require attention, but their mechanisms and interventions differ.

In this cohort, greater day +60 weight loss was observed among patients with viral infections and prolonged hospitalization, while reduced calorie intake was associated with bacterial infections at multiple time points. These associations are biologically plausible. Poor nutritional reserve may contribute to immune dysfunction and has been associated with adverse HSCT outcomes, including GVHD, non-relapse mortality, infection-related complications, and poorer recovery [16-18]. Conversely, patients who develop infection, mucositis, diarrhea, GVHD, or systemic inflammation may experience reduced appetite, impaired absorption, increased metabolic demand, and greater catabolism. The relationship is therefore likely bidirectional. The small subgroup sizes at day +60 also require caution. These results should be used to strengthen clinical surveillance and generate hypotheses for larger studies rather than to infer direct causation.

The linear mixed-effects model adds value by accounting for the repeated-measures structure of the data. The model showed that age, sex, and diagnosis were not statistically significant fixed-effect predictors of post-transplant weight change. The low marginal R-squared value suggests that these measured demographic and diagnostic covariates explained little of the observed variation. In contrast, the conditional R-squared value and intraclass correlation coefficient indicate that patient-level and time-related heterogeneity captured a much larger share of weight-change variability. This does not mean that weight loss is independent of all clinical factors, nor does it establish weight loss as a causal driver of outcomes. Rather, it suggests that nutritional decline is a common dynamic event during HSCT and should be monitored directly instead of being assumed from baseline diagnosis, age, or sex alone.

The clinical implications are practical. HSCT recipients require nutritional assessment before conditioning, close inpatient dietetic monitoring, safe and culturally acceptable food options, early oral nutritional supplementation, and timely escalation to enteral or parenteral nutrition when oral intake remains inadequate [5,19]. Post-discharge follow-up should include weight recovery, appetite, gastrointestinal symptoms, functional status, and quality of life [19-21]. Protein intake and body composition assessment would further improve future nutritional evaluation, particularly because weight alone may be confounded by fluid status and inflammation.

This study has limitations. It was conducted at a single center with 50 patients, which limits statistical power and introduces potential selection bias. The ambispective design resulted in differences in data completeness. Later time points had fewer available observations, leading to potential survivorship bias and missing-data bias, particularly if missingness was related to clinical deterioration, early discharge, or death. Calorie intake estimation relied heavily on dietary recall and retrospective record review, which introduces both recall bias and measurement bias, especially because documentation was not prospectively standardized for all patients. Protein intake was not reliably available and therefore could not be analyzed. The cohort was clinically heterogeneous, combining pediatric and adult patients, benign and malignant disorders, and allogeneic and autologous transplants. This heterogeneity improves real-world relevance but limits causal interpretation and may obscure age-specific or disease-specific nutritional trajectories. Quality-of-life assessment and longer-term nutritional recovery could not be evaluated in depth. Larger multicenter prospective studies with standardized nutritional definitions, protein-intake measurement, body composition assessment, predefined intervention pathways, and longer follow-up are needed.

## Conclusions

Peri-transplant nutritional decline was common in this Indian HSCT cohort. Weight loss progressed after transplant and persisted into early follow-up, while calorie intake declined sharply in the first week and then improved gradually. Greater weight loss and reduced calorie intake were associated with selected adverse outcomes, particularly infections and longer hospitalization, although the observational design does not establish causality.

These findings support routine integration of structured nutritional assessment and proactive dietetic care into HSCT practice. Early identification of nutritional risk, serial inpatient monitoring, timely nutritional supplementation, and post-discharge nutrition-focused follow-up may help improve recovery and reduce morbidity. Larger prospective multicenter studies are required to confirm these associations and define standardized nutrition-focused interventions for HSCT recipients.

## References

[1] de Defranchi RLB, Bordalejo A, Cañueto I, Villar A, Navarro E (2015). Evolution of nutritional status in patients with autologous and allogeneic hematopoietic stem cell transplant. Support Care Cancer.

[2] Eglseer D, Bauer S, Huber-Kraßnitzer B, Greinix H (2021). Malnutrition risk prior to hematopoietic stem cell transplantation predicts mortality in adults. Bone Marrow Transplant.

[3] El-Ghammaz AM, Matoug RB, Elzimaity M, Mostafa N (2017). Nutritional status of allogeneic hematopoietic stem cell transplantation recipients: influencing risk factors and impact on survival. Support Care Cancer.

[4] Biswas B, Naskar NN, Basu K, Dasgupta A, Basu R, Paul B (2021). Malnutrition, its attributes, and impact on quality of life: an epidemiological study among β-thalassemia major children. Korean J Fam Med.

[5] Fuji S, Einsele H, Savani BN, Kapp M (2015). Systematic nutritional support in allogeneic hematopoietic stem cell transplant recipients. Biol Blood Marrow Transplant.

[6] Sorror ML, Maris MB, Storb R, Baron F, Sandmaier BM, Maloney DG, Storer B (2005). Hematopoietic cell transplantation (HCT)-specific comorbidity index: a new tool for risk assessment before allogeneic HCT. Blood.

[7] National Institute of Nutrition - Indian Council of Medical Research (2011). Dietary Guidelines for Indians: A Manual.

[8] (2017). Common Terminology Criteria for Adverse Events (CTCAE), Version 5.0. https://dctd.cancer.gov/research/ctep-trials/for-sites/adverse-events/ctcae-v5-8x11.pdf.

[9] Harris AC, Young R, Devine S (2016). International, multicenter standardization of acute graft-versus-host disease clinical data collection: a report from the Mount Sinai Acute GVHD International Consortium. Biol Blood Marrow Transplant.

[10] Aaronson NK, Ahmedzai S, Bergman B (1993). The European Organization for Research and Treatment of Cancer QLQ-C30: a quality-of-life instrument for use in international clinical trials in oncology. J Natl Cancer Inst.

[11] von Elm E, Altman DG, Egger M, Pocock SJ, Gøtzsche PC, Vandenbroucke JP (2008). The strengthening the reporting of observational studies in epidemiology (STROBE) statement: guidelines for reporting observational studies. J Clin Epidemiol.

[12] Rieger CT, Wischumerski I, Rust C, Fiegl M (2015). Weight loss and decrease of body mass index during allogeneic stem cell transplantation are common events with limited clinical impact. PLoS One.

[13] Fuji S, Mori T, Khattry N (2015). Severe weight loss in 3 months after allogeneic hematopoietic SCT was associated with an increased risk of subsequent non-relapse mortality. Bone Marrow Transplant.

[14] Miller LJ, Halliday V, Snowden JA, Aithal GP, Lee J, Greenfield DM (2024). Health professional attitudes and perceptions of prehabilitation and nutrition before haematopoietic cell transplantation. J Hum Nutr Diet.

[15] Vieira IB, Sette NS, de Oliveira CA, Correia MI, Duarte CK, Generoso SV (2024). Comparison of pre- and posttransplant energy expenditure in patients undergoing hematopoietic stem cell transplantation and evaluation of associated factors. Nutrition.

[16] Hirose EY, de Molla VC, Gonçalves MV (2019). The impact of pretransplant malnutrition on allogeneic hematopoietic stem cell transplantation outcomes. Clin Nutr ESPEN.

[17] Khosroshahi RA, Barkhordar M, Talebi S, Imani H, Sadeghi E, Mousavi SA, Mohammadi H (2023). The impact of malnutrition on mortality and complications of hematopoietic stem cell transplantation in patients with acute leukemia. Clin Nutr.

[18] Morello E, Guarinoni MG, Arena F (2020). A systematic review of the literature and perspectives on the role of biomarkers in the management of malnutrition after allogeneic hematopoietic stem cell transplantation. Front Immunol.

[19] Estran S, Loschi M, Benachour S (2024). Improving nutritional status after allogeneic stem cell transplantation: results of phase 2 ALLONUT clinical trial. Bone Marrow Transplant.

[20] Grulke N, Albani C, Bailer H (2012). Quality of life in patients before and after haematopoietic stem cell transplantation measured with the European Organization for Research and Treatment of Cancer (EORTC) Quality of Life Core Questionnaire QLQ-C30. Bone Marrow Transplant.

[21] Cioce M, Botti S, Lohmeyer FM (2022). Nutritional status and quality of life in adults undergoing allogeneic hematopoietic stem cell transplantation. Int J Hematol.

